# Correlation between impedance cardiography and 6 min walk distance in atrial fibrillation patients

**DOI:** 10.1186/s12872-016-0297-0

**Published:** 2016-06-10

**Authors:** Ling Ding, Xiao-Qing Quan, Shu Zhang, Lei Ruan, Le Zhang, Kai Zheng, Wei-Wei Yu, Xiao-Fen Wu, Tao Mi, Cun-Tai Zhang, Hong-Lian Zhou

**Affiliations:** Department of Geriatrics, Tongji Hospital, Tongji Medical College, Huazhong University of Science and Technology, Wuhan, 430030 China; Department of Cardiology, Tongji Hospital, Tongji Medical College, Huazhong University of Science and Technology, Wuhan, 430030 China

**Keywords:** Impedance cardiography, 6 min walk distance, Atrial fibrillation

## Abstract

**Background:**

The correlation between impedance cardiography (ICG) and 6 min walk distance (6MWD) in atrial fibrillation (AF) patients remains unknown.

**Methods:**

We recruited 49 subjects in the study (21 AF patients and 28 patients without AF) and estimated hemodynamic parameters: cardiac output (CO), stroke volume (SV), stroke volume index (SVI), left stroke work (LSW), left stroke work index (LSWI), stroke systemic vascular resistance (SSVR), stroke systemic vascular resistance index (SSVRI); 6MWD, left ventricle ejection fraction (LVEF), NT-pro brain natriuretic peptide (NT-pro BNP) for the two groups.

**Results:**

The AF group have apparently lower CO (2.26 ± 0.14 VS 4.11 ± 0.20 L/min, *p* = 0.039) and distinctly higher SVR (677.60 ± 69.10 VS 344.41 ± 22.98 dynes/cm^5^, *p* = 0.001), SSVRI (396.97 ± 36.80 VS 199.01 ± 11.72 dynes/cm^5^/m^2^, *p* < 0.001) than the control group. NT-pro BNP (1409.48 ± 239.90 VS 332.59 ± 68.85 pg/ml, *p* = 0.001) in the AF group was significantly higher than the control group and 6MWD (264.33 ± 14.55 VS 428.79 ± 29.98 m, *p* < 0.001) in the AF group was lower than the control group. There was no significant difference in LVEF between the two groups (62.67 ± 7.62 % VS 63.93 ± 5.03 %, *p* = 0.470). Pearson correlation analysis revealed that CO (R = 0.494, *p* = 0.023), SV (R = 0.633, *p* = 0.002), LSW (R = 0.615, *p* = 0.003) and LSWI (R = 0.491, *p* = 0.024) significantly correlated positively with 6MWD in AF patients.

**Conclusions:**

AF patients had lower cardiac output, shorter 6MWD and higher NT-pro BNP than patients with sinus rhythm. The cardiac output measured by impedance cardiography significantly correlated positively with 6MWD in AF patients.

## Backgrounds

Atrial fibrillation (AF) is a kind of common cardiac arrhythmia [1], which disrupts the movement of the left atrial. AF is defined as an irregular, disorganized electrical activity of the atria and it is a growing clinical problem associated with increased morbidity and mortality [2, 3]. Estimating cardiac function in AF patients contributes to judge prognosis and adjust therapeutic schedule. Traditionally, left ventricle ejection fraction (LVEF), the fraction of outbound blood pumped from the heart with each heartbeat measured by cardiac color ultrasound (CCU) has been chosen to assess the cardiac function in patients including AF. However, stroke volume changed in different cardiac cycles because of irregular diastole and systole of the atria [3, 4]. Lacking of an active contraction of the LA (atrial kick) in late diastole, cardiac output in AF patients may be less than in normal persons. LVEF measured by CCU only reflect cardiac function of contraction in one cardiac cycle, not the whole cardiac function in AF patients.

A considerable proportion of previous data have stated the role of hemodynamic parameters measured by impedance cardiography (ICG) in diagnostic, prognostic, and therapeutic decision making in chronic heart failure [5–7] and pulmonary hypertension [8]. ICG is a reliable, noninvasive technique that can be easily used to continually measure hemodynamic parameters and output the mean values [9]. ICG is based on the Ohm’s law stating that when a constant current travels through a conductor, changes in voltage are directly proportional to variations in impedance [9]. Bio-impedance decreases across the chest during systole as a result of an increase in aortic blood volume and flow velocity. There are series of hemodynamic parameters associated with cardiac function such as cardiac output (CO), stroke volume (SV), stroke volume index (SVI), left stroke work (LSW), left stroke work index (LSWI), stroke systemic vascular resistance (SSVR) and stroke systemic vascular resistance index (SSVRI) measured by ICG.

Gilewski et al. investigated cardiovascular changes in tissue Doppler echocardiography (TDE) and ICG in AF patients subjected to cardioversion [10]. The study suggest that ICG seems to be complementary to standard echocardiography, and to some extent to surpass TDE. However, there is little published study on the correlation between ICG and 6 min walk distance (6MWD) or the value of ICG in predicting the cardiac function in AF patients. Although AF patients have irregular diastole period, ICG could continually measure parameters related to cardiac function, which provides more accurate information to predict the cardiac condition in AF patients. The present study will research the values of impedance cardiography in predicting cardiac function and the correlation between hemodynamic parameters and 6MWD in AF patients.

## Methods

### Study sample

This study was approved by the Clinical Research Ethics Committee of Tongji Hospital, Tongji Medical College, Huazhong University of Science and Technology. All participating persons were given informed consent and written informed consent was obtained from all subjects before enrollment. The study was performed between January 2014 and December 2015. We recruited patients with AF (*n* = 21) and healthy controls (*n* = 28). All the AF patients in our study were persistent or longstanding persistent AF according to 2014 AHA/ACC/HRS guideline [11]. Patients in healthy control group were sinus rhythm confirmed by electrocardiogram and ambulatory electrocardiogram. Patients in the two groups were matching for age, gender, height, weight, body mass index (BMI), underlying disease and medicine.

Exclusion criteria were impaired cognition, chronic obstructive pulmonary diseases (COPD), hyperthyroidism, severe hepatic disease, severe renal impairment, asthma, cystic, fibrosis, arthritis, ankle, knee or hip injuries, muscle wasting, any life-threatening disease, drugs and/or alcohol abuse [12, 13]. Additionally, we excluded patients with pacemaker implantation, valvular heart disease, enlarged LV dimension, recent myocardial infarction, unstable angina, candidacy for revascularization, cardiomyopathy, congestive heart failure, pulmonary heart disease, a resting heart rate of more than 120 bpm, a systolic blood pressure of more than 180 mmHg, and a diastolic blood pressure of more than 100 mmHg [10, 12, 13].

### Data collection

ICG was performed using the Cheer Sails Medical (CSM3000 system) with electrodes placed on the neck and in the hypochondriac regions. The basic principle of this methodology is that variations in the impedance (Z) to an alternating high-frequency (75KHz) and low magnitude (1.8 mA) current, across the thorax during cardiac ejection, result in a specific waveform that can be used to calculate stroke volume [14]. After 5 min of rest in the supine position in an examination room, a technician performed ICG. The hemodynamic variables CO, SV, SVI, LSW, LSWI, SVR, and SVRI referring to the cardiac function and health condition were showed. Values of the hemodynamic parameters represented the mean value over the 3-minute sampling period.

CCU was performed with the aim of measuring LVEF. Diastolic left ventricular diameter (DLVD), left ventricular posterior wall thickness (LVPWT), interventricular septal thickness (IVST) and left atrial diameter (LAD) were also recorded. The 6MWT was performed according to standard guideline [12]. A single walk test without practice was administered. The 6 min walk test (6MWT) was ordered by the patient’s physicians as an initial or follow-up assessment of their cardiac function according to Automatic Test System (ATS) standards [12, 13]. All distance data were recorded accurately. NT-pro BNP were tested by picking venous blood in clinical laboratory.

### Data analysis

Baseline subject characteristics across quartiles were compared using independent-samples t test for continuous variables and Pearson χ^2^ test for dichotomous variables [15, 16]. All the statistic values of continuous variables was mean ± standard error. In χ^2^ test, continuity correction was for dichotomous variables with fewer than 5 participants in a categories. Statistical significance was set to a p value less than 0.05. The strength of association between variables was performed using Pearson correlation test [17].

## Results

Baseline characteristics of the AF group and the control group were presented in Table 1. The subjects in the two groups had no statistical difference in age, gender, weight, BMI, or underlying disease such as diabetes mellitus and hypertension or basic medicine such as β-receptor blocker. There were five (23.8 %) patients in the AF group using digoxin and nobody in the control group using the same medicine, which made statistical difference in the two groups.

**Table 1:** Baseline characteristics of all the subjects

Parameters	Control group (*n* = 28)	AF group (*n* = 21)	t/χ^2^	*P*
Age (years)	75.36 ± 2.23	83.19 ± 2.0	2.523	0.093
Gender, n (% Male)	20 (71.4)	17 (81.0)	0.589	0.443
Height (cm)	166.07 ± 1.79	164.76 ± 1.47	−0.540	0.043
Weight (kg)	65.25 ± 2.184	61.57 ± 2.260	−1.152	0.421
BMI (kg^2^/m)	23.52 ± 0.52	22.68 ± 0.79	−0.929	0.191
Heart rate (bpm)	68.50 ± 1.99	68.90 ± 2.24	0.135	0.890
SBP (mmHg)	120.57 ± 2.91	120.43 ± 3.83	−0.030	0.676
DBP (mmHg)	67.25 ± 1.73	69.00 ± 1.63	0.715	0.279
Hypertension, n (%)	20 (71.4)	18 (85.7)	0.706	0.401
T_2_DM, n (%)	4 (14.3)	3 (14.3)	0	1.000
CRD, n(%)	2(7.1)	1(4.8)	0.118	1.000
CAD, n(%)	4(14.3)	2(9.5)	0.253	0.688
β-blocker treatment, n (%)	8 (28.6)	7 (33.3)	0.128	0.720
Digoxin treatment, n (%)	0 (0)	5 (23.8)	5.053	0.025

*Abbreviations: BMI* body mass index, *SBP* systolic blood pressure, *DBP* diastolic blood pressure*, T2DM* type 2 diabetes mellitus, *CRD* chronic renal disease, *CAD* coronary artery disease

Table 2 defined the comparison of hemodynamic parameters, 6MWD, LVEF and NT-pro BNP between the AF group and the control group. The present study found that the 6MWD in AF patients was shorter than the control group (264.33 ± 14.55 VS 428.79 ± 29.98 m, *p* < 0.001). The patients in the AF group had apparently lower CO (2.26 ± 0.14 VS 4.11 ± 0.20 L/min, *p* = 0.039) and distinctly higher SVR (677.60 ± 69.10 VS 344.41 ± 22.98 dynes/cm^5^, *p* = 0.001), SSVRI (396.97 ± 36.80 VS 199.01 ± 11.72 dynes/cm^5^/m^2^, *p* < 0.001) than those in the control group. NT-pro BNP (1409.48 ± 239.90 VS 332.59 ± 68.85 pg/ml, *p* = 0.001) in the AF group was significantly higher than the control group. There was no significant difference in LVEF between the two groups (62.67 ± 7.62 % VS 63.93 ± 5.03 %, *p* = 0.470). No significant difference was observed in other measured echocardiographic parameters (Table 2).

**Table 2:** Comparison of hemodynamic, 6MWD, LVEF, NT-pro BNP between the control group and the AF group

Parameter	Control group (*n* = 28)	AF group (*n* = 21)	t	*p*
ICG parameters				
CO (L/min)	4.11 ± 0.20	2.26 ± 0.14	4.502	0.039
SV (mL)	60.28 ± 3.30	32.45 ± 2.55	0.885	0.352
SVI (mL/m^2^)	34.93 ± 1.61	18.89 ± 1.74	0.086	0.771
SVR (dynes/cm^5^)	344.41 ± 22.98	677.60 ± 69.10	11.549	0.001
SVRI (dynes/cm^5^/m^2^)	199.01 ± 11.72	396.97 ± 36.80	14.556	<0.001
LSW (gm-m/beat)	66.42 ± 3.72	37.03 ± 2.77	3.429	0.070
LSWI (gm-m/m^2^/beat)	38.96 ± 1.85	22.42 ± 1.59	2.421	0.126
CCU parameters				
LVEF (%)	64.57 ± 0.92	63.05 ± 1.84	3.307	0.075
LAD (mm)	33.86 ± 1.00	40.67 ± 1.71	3.634	0.164
DLVD (mm)	44.96 ± 0.72	46.67 ± 1.44	1.140	0.056
IVST (mm)	10.21 ± 0.24	9.95 ± 0.24	0.781	0.586
LVPWT (mm)	9.89 ± 0.22	9.75 ± 0.20	0.489	0.981
Other parameters				
NT-pro BNP (pg/ml)	332.59 ± 68.85	1409.48 ± 239.90	13.470	0.001
6MWD (m)	428.79 ± 29.98	264.33 ± 14.55	16.816	<0.001

*Abbreviations: AF* atrial fibrillation, *ICG* impedance cardiography, *6MWD* 6 min walk distance, *LVEF* left ventricle ejection fraction, *NT-pro BNP* plasma NT-pro brain natriuretic peptide, *CO* cardiac output, *SV* stroke volume, *SVI* stroke volume index, *SSVR* stroke systemic vascular resistance, *SSVRI* stroke systemic vascular resistance index, *LSW* left stroke work, *LSWI* left stroke work index, *CCU* cardiac color ultrasound*, LAD* left atrial diameter, *DLVD* diastolic left ventricular diameter, *IVST* interventricular septal thickness, *LVPWT* left ventricular posterior wall thickness

As shown in Table 3, Pearson correlation analysis revealed that CO (R = 0.494, *p* = 0.023), SV (R = 0.633, *p* = 0.002), LSW (R = 0.615, *p* = 0.003) and LSWI (R = 0.491, *p* = 0.024) significantly correlated positively with 6MWD. LVEF did not significantly correlate with 6MWD (R = 0.037, *p* = 0.803).

**Table 3:** Correlation between hemodynamic parameters, LVEF and 6MWD in all patients

Parameters	Control group R	*p*	AF group R	*P*
CO (L/min)	0.626	<0.001	0.494	0.023
SV (mL)	0.587	0.001	0.633	0.002
SVI (mL/m^2^)	0.518	0.005	0.512	0.018
SVR (dynes/cm^5^)	−0.261	0.179	−0.331	0.143
SVRI (dynes/cm^5^/m^2^)	−0.389	0.041	−0.413	0.063
LSW (gm-m/beat)	0.488	0.008	0.615	0.003
LSWI (gm-m/m^2^/beat)	0.488	0.008	0.491	0.024
LVEF (%)	0.106	0.591	−0.307	0.176
NT-pro BNP (pg/ml)	−0.503	0.006	0.378	0.091

*Abbreviation: R* Correlation coefficient

## Discussion

In previous published study, non-invasive impedance cardiography has been used to reveal heterogeneity of hemodynamic parameters in primary hypertension [18, 19] and guide antihypertensive therapy [19–21]. However, there is no published data studying the role of impedance cardiography in patients with atrial fibrillation or the correlation between hemodynamic parameters and 6MWD. In the present study, we explored the feature of hemodynamic parameters in AF patients and statistical correlation among hemodynamic parameters, LVEF, NT-pro BNP and 6MWD.

6MWT is used to measure the distance that a patient can quickly walk on a flat, hard surface in a period of 6 min [12], and 6MWD is the distance in 6MWT. Due to wide availability, security and ease of implementation, 6MWT had been used in the assessment of functional capacity, evaluating exercise tolerance, prognosis and therapeutic effectiveness in patients with impaired cardiac function [22–27]. The present study found that 6MWD in AF patients was shorter than the control group (264.33 ± 14.55 VS 428.79 ± 29.98 m, *p* < 0.001) whereas LVEF had no significant difference between the AF group and the control group (62.67 ± 7.62 % VS 63.93 ± 5.03 %, *p* = 0.470). Our study also found that LVEF did not correlate with 6MWD (R = 0.037, *p* = 0.803). The result suggested that LVEF could not predict cardiac function accurately in AF patients.

BNP levels could be higher in patients with impaired cardiac function [26, 28]. The present data showed that AF patients had significantly higher NT-pro BNP than the control group (1385.74 ± 1102.51 VS 404.54 ± 322.33 pg/ml, *p* < 0.001). Although the value of LVEF in AF patients remained normal in the present study, the cardiac function in the AF group was worse than the control group according to the 6MWD and NT-pro BNP.

Due to great reliability and validity, ICG had been used in the diagnosis, prognosis and therapy in a variety of diseases [20, 21]. A recent study of AF patients subjected to cardioversion suggests that ICG seems to be complementary to standard echocardiography, and to some extent to surpass TDE [10]. The present study discovered that the CO (2.26 ± 0.14 VS 4.11 ± 0.20 L/min, *p* = 0.039) measured by impedance cardiography in AF patients were lower than the control group. Our data also found that CO (R = 0.494, *p* = 0.023), SV (R = 0.633, *p* = 0.002), LSW (R = 0.615, *p* = 0.003) and LSWI (R = 0.491, *p* = 0.024) correlated positively with 6MWD. Lacking of the atrial kick in late diastole [4], the cardiac output in AF patients was lower than the non AF patients. With lower cardiac output, the AF patients generally have shorter 6MWD.

6MWD had been proved to be useful and reliable in the assessment of functional capacity, evaluating exercise tolerance, prognosis and therapeutic effectiveness in patients with impair cardiac function [22, 29, 30]. The present study detected that hemodynamic parameters had a significant correlation with the 6MWD whereas LVEF measurements correlated poorly with the 6MWD in AF patients. The results of this study suggest that hemodynamic parameters measured by ICG could provide more accurate information than LVEF to predict cardiac function in AF patients. It is noteworthy that all the AF patients in our study were persistent or longstanding persistent AF, and we excluded AF patients with obviously abnormal heart structure. This strengthen the novelty of the present study.

### Clinical implications

Digitalis and beta-blockers have long been used to control heart rate in AF patients in order to reduce AF-related symptoms [31, 32]. The main hemodynamic change following the treatment with beta-blockers is a decrease of cardiac output. Digoxin therapy could increase the cardiac output and offer symptomatic improvement in AF patients with decreased cardiac output and cardiac function [33, 34]. Therefore, evaluating the cardiac output and cardiac function in AF patients is important. Although the value of LVEF in AF patients remained normal in the present study, the cardiac function in the AF patients was worse than the control group according to the 6MWD and NT-pro BNP. Our study also found that cardiac output measured by ICG significantly correlated positively with 6MWD in AF patients, and LVEF did not correlate with 6MWD. Clinical doctors may adjust therapeutic schedule of digitalis and beta-blockers according to the information from ICG. This is just our speculation according to our finding and the specific application need further investigation.

### Study limitations

Our study has limitations: (1) The study sample was relatively small, limiting the statistical power of group analyses. (2) The subjects in our study were relatively old and their parameters maybe could not represent the condition of the whole population. (3) We did not provide some important echocardiographic parameters such as cardiac index and left ventricular end-diastolic volume. Whether the diastolic dysfunction contribute to the decreased cardiac output in AF patients is not clear. Further studies are needed to determine the mechanism underlying the hemodynamic disorders observed in AF.

## Conclusion

Compared to patients with sinus rhythm, AF patients had lower cardiac output, shorter 6MWD and higher NT-pro BNP. The cardiac output measured by impedance cardiography significantly correlated positively with 6MWD in AF patients.

## Abbreviations

6MWD, 6 min walk distance; 6MWT, 6 min walk test; AF, atrial fibrillation; ATS, Automatic Test System; BMI, body mass index; CCU, cardiac color ultrasound; CO, cardiac output; COPD, chronic obstructive pulmonary diseases; DLVD, diastolic left ventricular diameter; EDV, end-diastolic volume; ICG, impedance cardiography; IVST, interventricular septal thickness; LAD, left atrial diameter; LSW, left stroke work; LSWI, left stroke work index; LVEF, left ventricle ejection fraction; LVPWT, left ventricular posterior wall thickness; NT-pro BNP, NT-pro brain natriuretic peptide; SSVR, stroke systemic vascular resistance; SSVRI, stroke systemic vascular resistance index; SV, stroke volume; SVI, stroke volume index
